# Artifacts in pulse transit time measurements using standard patient monitoring equipment

**DOI:** 10.1371/journal.pone.0218784

**Published:** 2019-06-21

**Authors:** Frank C. Bennis, Carola van Pul, Jarno J. L. van den Bogaart, Peter Andriessen, Boris W. Kramer, Tammo Delhaas

**Affiliations:** 1 Department of Biomedical Engineering, Maastricht University, Maastricht, The Netherlands; 2 MHeNS School for Mental Health and Neuroscience, Maastricht University, Maastricht, The Netherlands; 3 Department of Clinical Physics, Máxima Medical Centre, Veldhoven, The Netherlands; 4 Department of Applied Physics, Eindhoven University of Technology, Eindhoven, The Netherlands; 5 Department of Medical and Information Technology, Máxima Medical Centre, Veldhoven, The Netherlands; 6 Department of Pediatrics, Máxima Medical Centre, Veldhoven, The Netherlands; 7 Department of Pediatrics, Maastricht University Medical Center, Maastricht, The Netherlands; 8 GROW School for Oncology and Developmental Biology, Maastricht University, Maastricht, The Netherlands; 9 CARIM School for Cardiovascular Diseases, Maastricht University, MD, Maastricht, the Netherlands; Cleveland Clinic, UNITED STATES

## Abstract

**Objective:**

Pulse transit time (PTT) refers to the time it takes a pulse wave to travel between two arterial sites. PTT can be estimated, amongst others, using the electrocardiogram (ECG) and photoplethysmogram (PPG). Because we observed a sawtooth artifact in the PTT while using standard patient monitoring equipment for ECG and PPG, we explored the reasons for this artifact.

**Methods:**

PPG and ECG were simulated at a heartrate of both 100 and 160 beats per minute while using a Masimo PPG post-processing module and a Philips patient monitor setup at the neonatal intensive care unit. Two different post-processing modules were used. PTT was defined as the difference between the R-peak in the ECG and the point of 50% increase in the PPG.

**Results:**

A sawtooth artifact was seen in all simulations. Both length (59.2 to 72.4 s) and amplitude (30.8 to 36.0 ms) of the sawtooth were dependent on the post-processing module used. Furthermore, the absolute PTT value differed up to 250 ms depending on post-processing module and heart rate. The sawtooth occurred because the PPG wave continuously showed a minimal prolongation during the length of the sawtooth, followed by a sudden shortening. Both artifacts were generated in the post-processing module containing Masimo algorithms.

**Conclusion:**

Post-processing of the PPG signal in the Masimo module of the Philips patient monitor introduces a sawtooth in PPG and derived PTT. This sawtooth, together with a large module-dependent absolute difference in PTT, renders the thus-derived PTT insufficient for clinical purposes.

## Introduction

**Fig 1:**
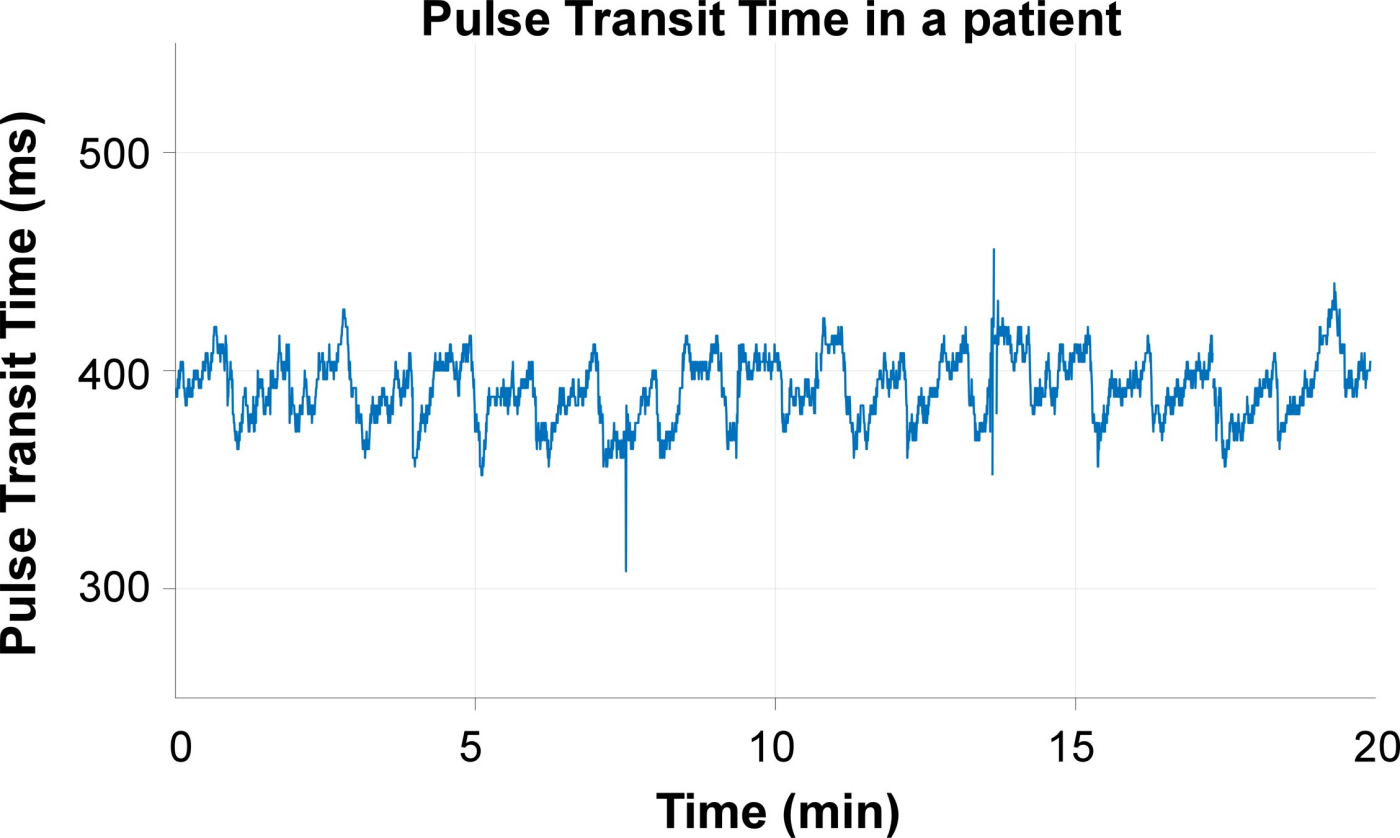
Clinically derived PTT of a preterm infant. A regular sawtooth-like artifact is seen, hampering clinical usefulness.

Pulse transit time (PTT) is commonly determined as the time difference between onset of cardiac ejection (approximated by the R peak in the electrocardiogram (ECG)) and the arrival of the pulse in a finger as determined from the photoplethysmogram (PPG) [1–4]. Increases in PTT are related to changes in the cardiovascular system such as lower systolic blood pressure, lower arterial stiffness or an increased pathway length [5–8]. PTT might be used, amongst others, to detect sleep disordered breathing [9] or as a surrogate measure of blood pressure due to the correlation of PTT with blood pressure [10]. Another promising application of PTT is monitoring of closure of the ductus arteriosus in a neonatal setting [11,12]. However, when we started our study on the applicability of PTT to detect the status of the ductus arteriosus, the estimated PTT showed a remarkable and relatively large sawtooth-like artifact (Fig 1). This artifact might not only impede clinical usefulness of PTT in the detection of a patent ductus arteriosus, but also the use of PTT as a surrogate measure of blood pressure because it reflects non-existing sudden changes in blood pressure. In the present study we explored why this sawtooth artifact occurred in PTTs obtained with standard patient monitoring equipment for ECG and PPG.

## Methods

**Fig 2:**
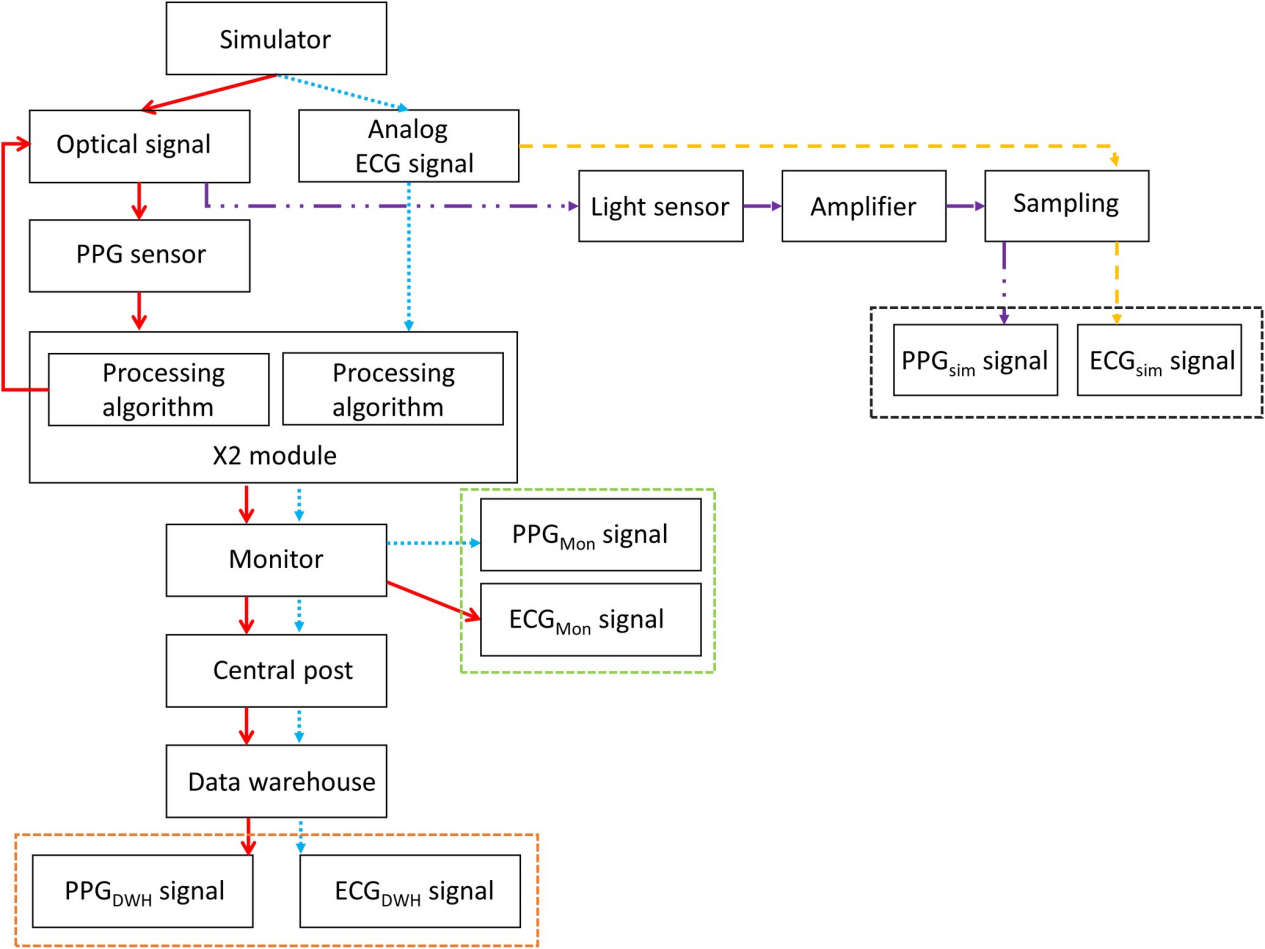
Flowchart of simulation setup. A simulator creates an analog ECG and an optical signal. The optical signal is measured with a PPG sensor and enters the X2 module together with the ECG for processing of the signal. The signal enters the monitor and is pushed to the central post and data warehouse. The signal can be extracted at the monitor (via RS232; green box) or is send to the central monitor and from there stored in a data warehouse and subsequently extracted (orange box). The optical signal and ECG are also measured directly from the output of the simulator, amplified, sampled and extracted (black box).

Experiments were performed at the neonatal intensive care unit at the Máxima Medisch Centrum in Veldhoven, The Netherlands. The Vital Signs Simulator ProSim 8 *(Fluke Biomedical*, *Cleveland*, *USA*) was used to create an artificial analog ECG output and an optical signal changing in intensity mimicking blood flow. The optical PPG sensor used *(Masimo SET SPO2‐technology*, *Irvine*, *USA)* has a light-emitting end as well as a light-receiving end. The light-emitting end offers pulsed light signals that are modulated due to the presence of blood in the tissue between the light-emitting and light-receiving ends of the sensor. These modulated signals are subsequently received by the light-receiving end. The intensity and duration of the light pulses are under control of the PPG post-processing module. In our setup, the simulator modulated the pulsed light signal in amplitude thereby mimicking a light signal that has passed through a finger with a pulsating blood flow. For a flowchart of the setup we refer to Fig 2. The optical PPG sensor as well as the ECG output were in turn connected to a post-processing module *(X2*, *Philips Medical Systems*, *Best*, *The Netherlands*). The post-processing module was connected to the patient monitor (*IntelliVue MX800*, *Philips Medical Systems*, *Best*, *The Netherlands*), which in turn was connected via the central post of the neonatal intensive care unit to a central data warehouse (*PIIC iX*, *Data Warehouse Connect*, *Philips Medical Systems*, *Best*, *The Netherlands*), where ECG and PPG data were stored at 250 and 125 Hz, respectively. The ECG and PPG were extracted for analysis from both the data warehouse and the monitor to test in which part of the chain potential artifacts occurred. Extracted data was stored on a personal computer (*Microsoft Windows 10 Pro*, *HP EliteBook 850 G3*, *Palo Alto*, *USA*). Simulations were performed at two different, but constant heart rates (100 and 160 beats per minute (BPM)) and processed by two different modules *(X2 A03 and X2 A05)*, resulting in a total of four simulations.

To exclude that the simulator itself or the light pulses offered by the PPG sensor introduced the sawtooth phenomenon in the PTT, a photosensitive diode was placed at the light-receiving end of the PPG sensor. The signal from this diode was amplified and sampled together with the ECG from the simulator at 25000 Hz *(Acquisition system IdeeQ*, *Instrumental Department Maastricht University*, *The Netherlands)*. The thus reconstructed PPG was smoothed over 5 samples to remove fluctuations. Estimation of PTT using these signals was done in the same manner as for the ECG and PPG from the data warehouse.

### PTT calculation

**Fig 3:**
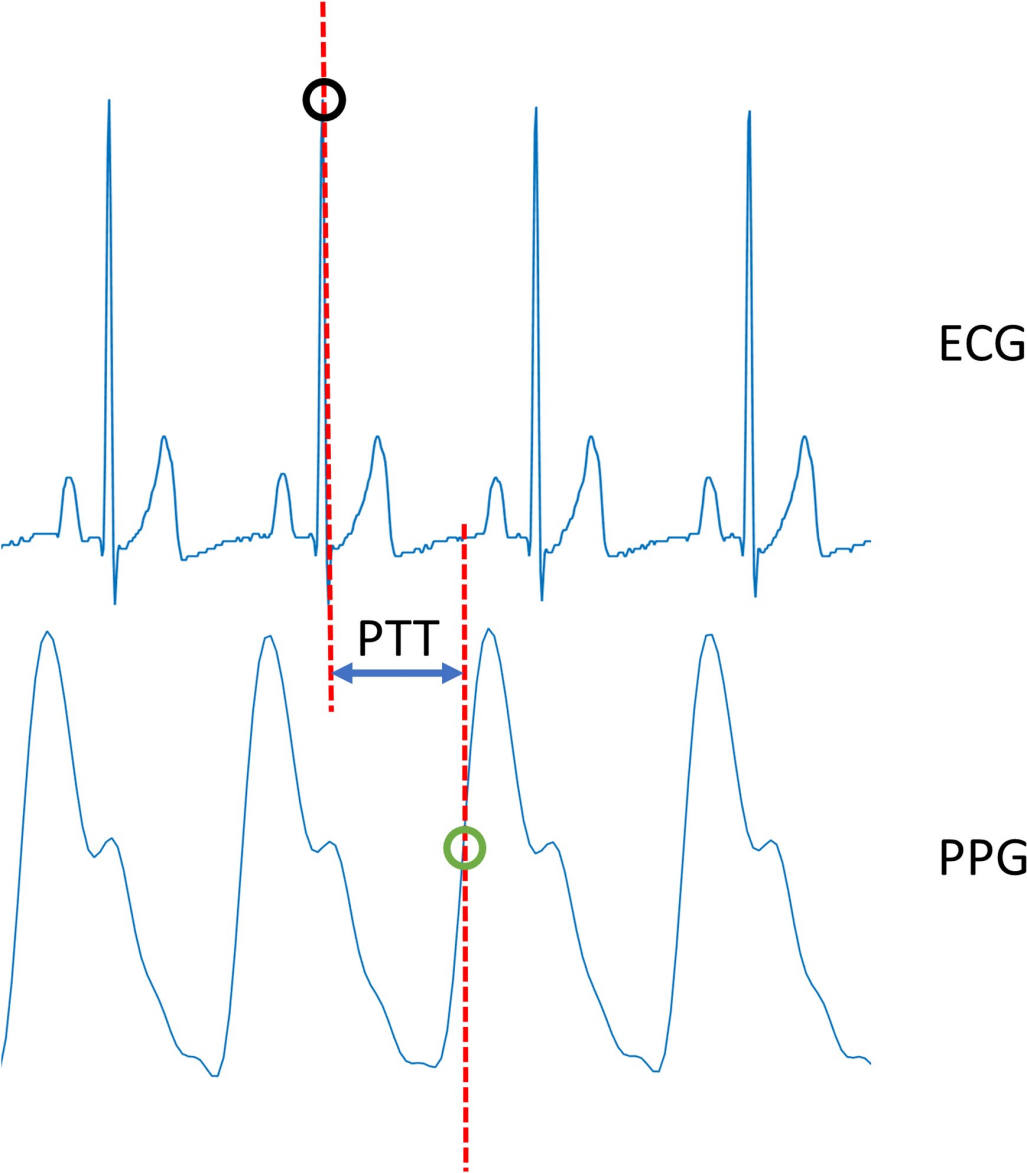
Illustration of pulse transit time calculation. Pulse transit time is depicted as the time difference between the R peak in the ECG (black circle) and the point of 50% increase in the PPG signal (green circle).

Signal analysis was performed using Matlab (*2016A*, *The MathWorks*, *Natick*, *MA*, *USA*). PTT was defined as the difference in time between the R peak in the ECG and the point where the increase in the upstroke of the PPG was 50% of the total increase (Fig 3). The R peak in the ECG was found using the Matlab build-in function *findpeaks*. The point of 50% increase in the PPG was selected by using the *findpeaks* function to find the lower and upper peak of the upstroke, followed by the selection of the first point where the increase was equal to or higher than 50%. The difference between the time instances for each ECG peak and subsequent PPG peak were calculated in milliseconds.

## Results

**Fig 4:**
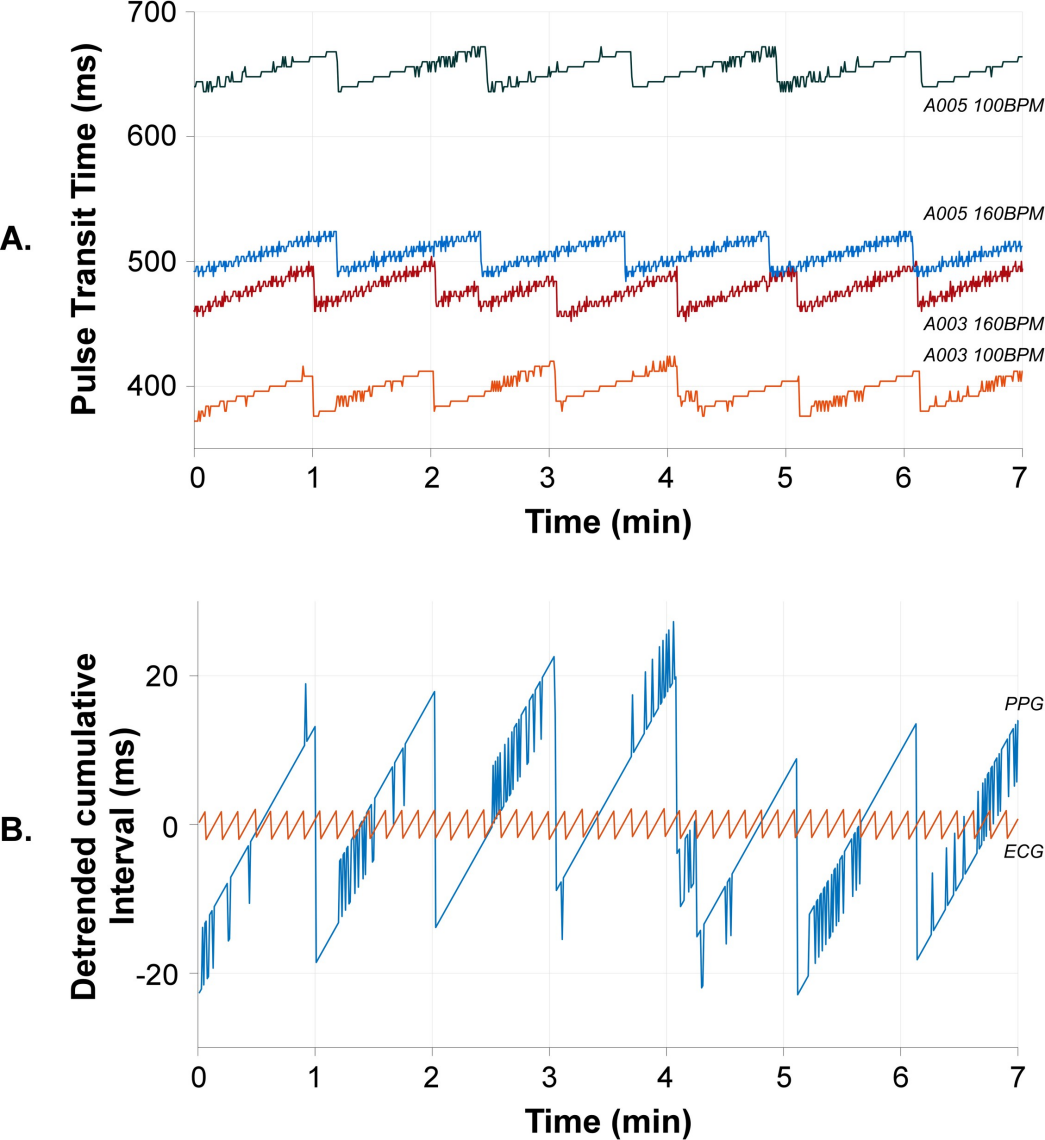
Pulse transit time and analysis for multiple simulations. **A.** Pulse transit time over several minutes for all four combinations of post-processing modules (A03 and A05) and heart rate (100 BPM and 160 BPM) using the Masimo oximeter. A clear sawtooth was observed in all simulations. **B.** Detrended cumulative intervals for PPG and ECG using the A03 module with 100 BPM. PPG shows a clear sawtooth pattern. The small fluctuations in ECG and PPG correspond to a single sample.

The PTT calculated for the A03 module (100 BPM signal in orange and 160 BPM in red) and the A05 module (100 BPM in black and 160 BPM in blue) are shown in Fig 4A. A recurring sawtooth-like phenomenon was detected in all simulations. The average length, slope and mean amplitude of the sawtooths are shown in Table 1 for all four simulations.

**Table 1:** Results of simulations.

	A03		A05	
	100 BPM	160 BPM	100 BPM	160 BPM
**Length (s)**	60.8	59.2	72.4	72.4
**Slope (ms/min)**	35.6	34.4	25.9	25.5
**Mean amplitude (ms)**	36.0	33.9	31.3	30.8

The length, slope and mean amplitude of an average sawtooth are shown for different heart rates (100 BPM and 160 BPM) with different post-processing modules (A03 and A05).

As the signal is simulated at a constant heart rate, the interval between subsequent R peaks or subsequent PPG peaks should remain equal. Therefore, the cumulative sum of these intervals over time should result in a straight upward line. Detrending this cumulative sum should result in a constant value of zero in case of a constant heart rate and stroke volume. The detrended cumulative intervals of the PPG and the ECG using the A03 module at 100 BPM are shown in Fig 4B. The detrended cumulative ECG interval did not show a slope, indicating that on average, every ECG complex had an equal length. However, the detrended cumulative PPG interval resembled the sawtooth as found in the PTT. This indicates that each waveform of the PPG signal was slightly prolonged, corrected for by a periodic shortening of a single waveform. The amount of prolongation and correction relate to the slope and the amplitude of the sawtooth in the PTT, respectively.

At similar heart rates but with different post-processing modules, PTT varied up to 250 ms, indicating a post-processing module dependent shift of the ECG and/or the PPG in time.

**Fig 5:**
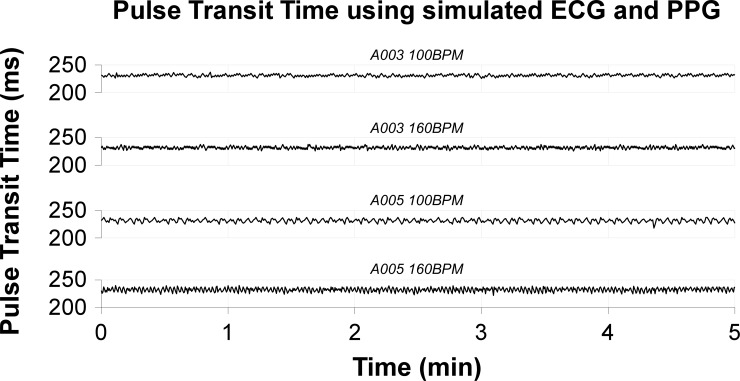
Pulse transit time as calculated from data extracted directly from the simulator. Pulse transit time over several minutes for all four combinations of post-processing modules (A03 and A05) and heart rate (100 BPM and 160 BPM) using direct extraction out of the simulator. No large sawtooth is observed. Small fluctuations are caused by shifting of detected peaks with one sample.

The PTT calculated from data directly extracted from the simulator did not show a sawtooth and was not influenced by heart rate or the module used to provide the optical signal (Fig 5). Because data extracted from the monitor and the data warehouse showed exactly the same sawtooth-like artifact, we conclude that the sawtooth artefact has its origin between the simulator and the monitor, i.e. it occurs in the post-processing module. This conclusion is supported by the module-dependent changes in the sawtooth characteristics.

## Discussion

We observed in our clinical monitor system a sawtooth pattern in the PTT, which has its origin in the Masimo post-processing module of the PPG signal in the Philips patient monitor. The detected sawtooth demonstrates that clinical patient monitoring systems need to be tested before they can be used for PTT measurements. Although direct export of the simulation signal did not show a sawtooth, this artifact was observed in the signal acquired directly at the patient monitor. Therefore, the sawtooth artifact is not a result of delays in signal transport between the patient monitor and the central post. Characteristics of this sawtooth were independent of the heart rate. However, the duration and slope of the sawtooth changed with different post-processing modules. This confirms that the sawtooth is generated in the post-processing module. Inspection of the individual ECG and PPG signals showed that the ECG did not show alterations in time, while all PPG waveforms were slightly prolonged, corrected for by a periodic shortening of a single waveform. Therefore, we conclude that postprocessing of the PPG waveform induces the sawtooth artifact. In addition to the sawtooth artifact, the change in PTT using different post-processing modules indicates that the ECG and/or the PPG are also shifted in time with a non-equal time interval. The results found in our study are in line with earlier observations by Foo et al., which describe a high variation in PTT using a Masimo oximeter [13]. Our study proves that the variation observed is actually a periodic artifact combined with a phase delay, both due to post-processing in the Masimo module.

We observed that the PTT_sim_ was constant over time and independent of HR and the post-processing module. However, the PTT_DWH_ showed a sawtooth with amplitude up to 36.0 ms and a shift in PTT values up to 250 ms, dependent on HR and the post-processing module used. Amirtharaj et al. found that the mean PTT for preterm infants with a closed ductus arteriosus is as small as 65.5 ms [11]. Even though in this study the PTT has a resolution of 8 ms, it is clear that artifacts as large as measured in the current setup result in a PTT that cannot be used as an absolute value in clinical situations and thus not as a diagnostic tool. The finding that the PTT is unreliable as a diagnostic tool has to be taken into account in current research, as current research focusses on the PTT as a predictive or trend parameter [10,14,15]. Our study emphasizes the importance of quality control in the setup used for measuring PTT.

To enable the use of PTT in a clinical setting, the cause of the phase shift and the sawtooth should be identified and removed by the manufacturer. An alternative is calibration of the signal based on a known input. We observed that the shift in PTT time and the sawtooth characteristics differ per post-processing module. As the shift in time is HR dependent and HR is not constant in patients, it is not possible to correct for this shift in time without knowing the exact nature of the dependencies. When the shift in time of the signals remains unknown, the absolute value of the PTT has no meaning. Therefore, the only clinical application of PTT is the use as a trend monitor. To use the PTT as a trend monitor, the sawtooth has to be corrected by a moving averaging filter with a window that spans the sawtooth artifact. This application is not able to detect beat-to-beat differences or to compare a patient to a reference value, as is necessary for predictive purposes. Therefore, for clinical applications of the PTT it is of utmost importance that both phase shift and sawtooth are prevented from occurring in the post-processing module.

Our study has several limitations. First, though we demonstrated that the Masimo post-processing module is responsible for the occurrence of the sawtooth, we cannot indicate why these artifacts occur because the algorithms used for post-processing are unknown to us. Therefore, we cannot provide solutions, but only cumbersome workarounds to use the current data to the fullest extent. Second, our methods to detect alterations in the time domain depend on raw waveform information, which is not always provided. Based on our findings, it is necessary to control if a sawtooth and a phase delay are present in the PTT. Therefore, systems that output the PTT without providing PPG- and ECG-signals should not be used.

## Conclusion

We demonstrated that post-processing of the PPG signal in the Masimo module of the Philips patient monitor introduces a sawtooth in the PPG and in the derived PTT. This sawtooth, together with a large module-dependent phase shift, renders the PTT insufficient for clinical purposes. Whether these artifacts are vendor-specific is unknown. We conclude that before using time-dependent parameters like PTT for clinical purposes, the monitoring system needs to be checked on its accuracy.

## Supporting information

S1 File Data direct from the simulator, module A03 at 100 BPM. (ZIP)

S2 File Data direct from the simulator, module A03 at 160 BPM. (ZIP)

S3 File Data direct from the simulator, module A05 at 100 BPM. (ZIP)

S4 File Data direct from the simulator, module A05 at 160 BPM. (ZIP)

S5 File Data measured by the Masimo module, all four combinations. (ZIP)
